# Does Environmental Interpretation Impact Public Ecological Flow Experience and Responsible Behavior? A Case Study of Potatso National Park, China

**DOI:** 10.3390/ijerph19159630

**Published:** 2022-08-05

**Authors:** Tiantian Tang, Minyan Zhao, Dan Wang, Xiangyu Chen, Wuqiang Chen, Chunwen Xie, Yan Ding

**Affiliations:** 1 College of Biodiversity Conservation, Southwest Forestry University, Kunming 650224, China; 2 Institute of Tibetan Plateau Research, Chinese Academy of Sciences, Beijing 100101, China; 3 School of International Chinese Language Education, Yunnan University, Kunming 650091, China; 4 School of Earth and Environmental Sciences, The University of Queensland, Brisbane, QLD 4072, Australia; 5 School of Economics and Management, Yunnan Forestry Technological College, Kunming 650224, China; 6 School of Geography and Ecotourism, Southwest Forestry University, Kunming 650224, China

**Keywords:** ecological flow experience, environmental interpretation, recreational preferences, recreational motivations, environmental education, environmentally responsible behavior, national park, Potatso

## Abstract

Being responsible for ensuring nature preservation, environmental interpretation raises people’s awareness of nature preservation as a form of public service, and enhance their environmentally responsible behavior. Based on the flow theory, this study proposes a conceptual model of environmental interpretation impacts on visit motivation, ecological experience, environmental attitudes, and environmental behaviors. Selecting the users (visitors) of environmental interpretation at Potatso National Park in Shangri-La, Yunnan Province, China, we obtained 568 valid questionnaires and used Amos software to analyze a structural equation model to verify the model. The results indicate that the environmental interpretation plays a significant role in enriching the public’s ecological experience, which is an intermediary variable in which visiting motivation influences environmental attitudes and guides environmentally responsible behavior. The research suggests that national parks should strengthen the environmental interpretation facilities experiential and available, and adjust the configuration of the existing interpretation media in the three-dimensional structure of theme, space, and time, considering the motivation of the public visits, enriching ecological experience, and inspiring service details.

## 1. Introduction

As an important part of China’s new system of natural protected areas, national parks have a social function to provide education about nature and an ecological experience. The ultimate goal of national parks is to promote nature preservation in protected areas. It is also importance to guide the public in responsible behavior and participation in nature preservation. National parks, as the main body in China’s natural protected areas system, would provide environmental interpretation services and activities to improve education and recreation function. Further clarifying the key points of the interaction among visit motivation, ecological experience, and environmental behaviors will provide a scientific basis for improving the nature preservation of national parks and the national well-being.

Environmental interpretation is a new comprehensive product in the field of international experience and local exploration; it is a way to communicate information between the supply side (national parks) and the demand side (the public). Free Tilden (1957) defined the environmental interpretation as an education activity that aims to bring meaning and relationship through use of original objectives, by firsthand experience with the resource or by illustrative media, rather than simply to communicate factual information [1]. Disseminating natural and humanistic knowledge to the public through both non-personnel and personnel media provides ecological flow experience to guide the public in acting responsibly [2]. The establishment of environmental interpretation occurred late in China, so the arrangement of facilities and services has not been sufficiently scientific, experiential, and participatory. The reason for this lies in the fact that more attention was paid to the external form rather than to the content. Most facilities are for display rather than for education and lack key technology for the management of public behavior [3]. As an important protected area, the development of environmental interpretation in national parks has become the focus of public participation in parks’ management and protection [4]. At present, the role of the public is actively changing from sightseers to participants, and as an auxiliary tool in this process, the status and role of environmental interpretation are extremely important [5]. However, most research on China’s environmental interpretation within national parks has focused on building an evaluation index system and studying public satisfaction, while most studies on ecological experience and responsible public behavior are simply qualitative descriptions, with little quantitative analysis. Published research discussing the relationship between and mechanisms of public ecological experience and environmentally responsible behavior is scarce, so there is a strong need to strengthen research in this area to provide a valuable reference for national park planning and policy making.

## 2. Research Review and Model Construction

### 2.1. Flow Theory

The American psychologist Csikszentmihalyi developed the concept of “flow”, which refers to the subjective experience of public action based on intrinsic motivation, which appears as high enjoyment, positivity, and all-out effort. The experience of flow can improve the quality of participation [6] and make public behavior more meaningful [7,8,9]. The theory of flow has been widely used in “experiential” industries [10], extending to education flow [11], and group flow [12]. Novak has suggested nine factors that create a positive result and cause flow: skill, control, challenge, arousal, telepresence, time distortion and exploratory behavior, focused attention, involvement, and interactivity [13]. Hamilton points out that the first five (external) factors that Novak suggests create flow, while the final four (internal) factors can be seen to represent the experience of flow [14]. Chinese experts have divided these factors into three categories: condition, experience, and result factors [10]. Many studies have shown that the condition of the flow is related to positive results [11,12,15], and products designed based on the theory of flow can create a better user experience and yield the best educational guidance effect [16,17].

**Figure 1:**
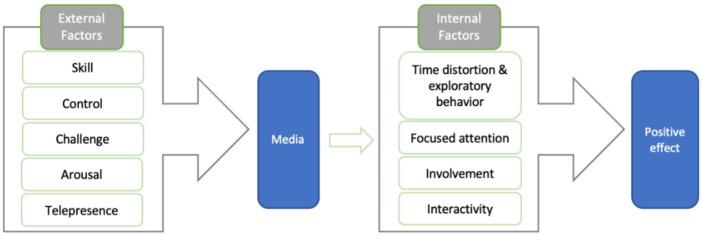
Flow theory.

This study uses the flow theory to explore the ecological flow experience that is created when external factors for flow are available and is further promoted by the media, which finally yields a positive result (see Figure 1). The public’s visit motivation corresponds to the external factors, the media refers to environmental interpretation, and the flow experience is produced by the internal factors resulting from the combined action of attitude and behavior. This flow experience promotes the generation of public ecological engagement and a positive environmental attitude, thus guiding the public in engaging in responsible behavior.

### 2.2. Hypothesis

As an external factor, environmental interpretation is a communication activity that is frequently used as an information channel. Before visiting the park, the public can get information about various elements of the national park, including natural geographical features (e.g., highland ecosystem) and human geographical features (e.g., nationality, culture, religion). Visitors may tend to be passive in their actual visit [18], so environmental interpretation can play an important role in creating information exchange with visitors and arousing their interest. Important measures of an ecological flow experience include enjoying the beautiful scenery, satisfying one’s curiosity, and gaining new knowledge. After visiting a national park, ecological experiences can lead the public to pay attention to environmental issues, support public reflection on these issues, and eventually lead to responsible behaviors [19]. Studies on flow consider different factors, and the interaction between an ecological experience guided by environmental interpretation and responsible behavior is weak. Studies on the interaction of the public’s national park visit motivation and responsible behavior remain limited, so further studies are needed to test the validity and necessity of incorporating environmental interpretation in national parks.

Based on the theory of flow, this paper explores ecological experience and outlines the hypotheses developed in the following sections.

#### 2.2.1. Visit Motivation Enhances the Validity of Environmental Interpretation

Motivation is the factor that prompts the public to visit a place. Based on Dann’s push–pull theory, the wish to visit is an internal driving factor (push), while the destination is an external attraction (pull), and the interaction between the two forms the push-pull motivation [20]. Evasion and pursuit are another two motivating factors [21]. There are seven types of socio-psychological push motivation, including escaping from a mundane environment, self-discovery and self-evaluation, relaxation, prestige, return, enhancing parental relationships, and enhancing one’s social network. In addition to these, pull motivations can be driven by factors like curiosity, education [22]; less pollution, noise, and pressure [23]; enjoying the peace and quiet [24]; and spiritual and regional needs [25]. Scholars have suggested that emotional communication [5], personal hobbies and networking, being close to nature and relaxing [26], and gaining friendship and knowledge [27] are the main reasons for the public to travel. Additional sources of motivations can be focused on spiritual and cultural aspects, adventure, leisure [28], literature [29], film [30] and the arts [31], while a child’s education is often the most important motivation in parent–child tourism.

Summarizing the previous literature and using the extended model of flow theory, the internal will to visit motivates the public to take part in environmental interpretation, which satisfies their curiosity and provides new knowledge. Willingness to participate and frequency of interaction therefore increase. Enjoyment includes a sense of freshness or accomplishment, and when one’s expectations are achieved or surpassed, the experience of flow occurs. Deep involvement promotes ecological experiences that lead to a change in perception and positive outcomes. Accordingly, we developed Hypothesis 1 and 2:

**Hypothesis** **1** **(H1).** *Visit motivation will have a positive effect on ecological flow experience.*

**Hypothesis** **2** **(H2).** *Visit motivation will have a positive effect on responsible behavior.*

#### 2.2.2. Environmental Interpretation Promotes the Ecological Flow Experience

The ecological experience is an important component that could further influence memory, including nature knowledge, local culture, entertainment, and relaxation [32,33]. There is already agreement on the importance of connecting resources and the public’s psychological experience, and environmental interpretation is trying to connect the two in different ways, which is an important way for the public to enhance cognition, enrich their ecological experience, and improve satisfaction. A satisfactory ecological experience greatly enhances public perception [27], and public perception directly influences responsible behavior. Higher satisfaction, perception, and participation could promote a public sense of environmental protection and lead to responsible behavior [28]. Environmental interpretation is used to deepen the public’s perception and appreciation of natural and socio-cultural resources, as well as improving their experience and influencing their thinking and behavior [29,30]. Environmental interpretation can be regarded as the core of leisure system planning, as it enriches the ecological experience [31], promotes a stable ecological ethical concept, and influences behavior [34]. Based on this idea, Hypothesis 3 is proposed:

**Hypothesis** **3** **(H3).** *Ecological flow experience will have a positive influence on responsible behavior.*

#### 2.2.3. Ecological Flow Experience Improves Environmental Attitude

Environmental attitude is a psychological concept [35] that reflects an individual’s concern for the environment, dependence on terrestrial resources, and humanity’s behavior and power to transform the environment [36]. Effective environmental interpretation adds an emotional component to interpretation, making it possible for the public to be satisfied with the landscape learning experience and building an emotional connection with the landscape, which leads to local attachment and a desire to ensure environmental protection [37,38], thus raising awareness of resource conservation [39]. This educational function improves the public’s thinking and the quality of ecological experience, as well as effectively leading and managing public behavior [40,41]. Based on these ideas, Hypothesis 4 is proposed:

**Hypothesis** **4** **(H4).** *Ecological flow experience will have a positive effect on environmental attitude.*

#### 2.2.4. Environmental Attitude Promotes Responsible Behavior

According to the theory of reasoned action, attitudes can consciously influence individual behavior. After participating in an environmental interpretation activity, 79% of the public have a positive attitude change, and responsible behavior can be seen in 50% of them [42]. This suggests that focused interpretation can effectively change attitudes and intentions related to protective behavior [43]. The environmental attitude involves belief and emotion [44], which makes it a long-term psychological reaction. Environmental attitude can, to some extent, affect responsible behavior [45]. Accordingly, Hypothesis 5 is put forward:

**Hypothesis** **5** **(H5).** *Environmental attitude will have a positive effect on responsible behavior.*

#### 2.2.5. Ecological Flow Experience Mediates Motivation, Attitude, and Behavior

Environmental interpretation can enrich the ecological flow experience and have a positive effect on environmental attitudes, while also decreasing adverse environmental behaviors [46,47]. The ecological flow experience is affected by environmental interpretation media, how well the interpretation content is understood, and the interaction between environmental attitude and willingness to engage in responsible behavior [3]. The interpretative content related to the environmental experience could indirectly affect the public’s environmental attitude and behavioral tendency, which confirms that environmental interpretation services can promote the development of ecological experience [48,49,50,51,52]. Thus, two hypotheses are put forward:

**Hypothesis** **6** **(H6).** *Ecological flow experience will mediate the influence of visiting motivation on responsible behavior.*

**Hypothesis** **7** **(H7).** *Ecological flow experience will mediate the influence of visiting motivation on environmental attitudes and guiding responsible behaviors.*

### 2.3. Conceptual Structure Model

**Figure 2:**
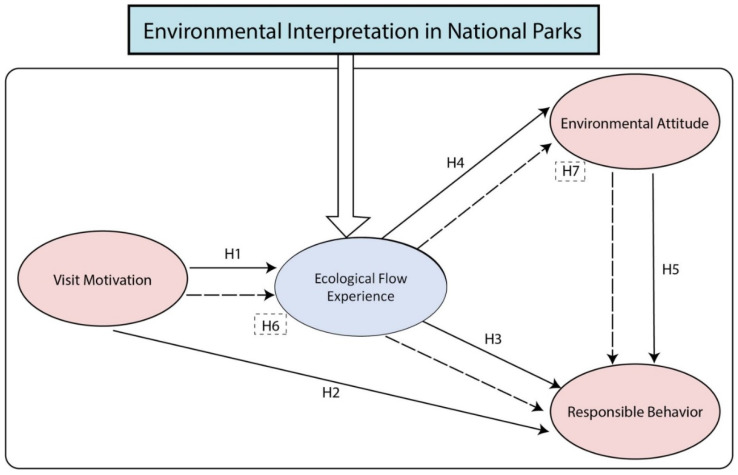
Conceptual model.

Based on a review of the relevant literature and flow theory, we consider four variables—visiting motivation, ecological flow experience, environmental attitude, and responsible behavior—under the premise that environmental interpretation in a national park is in effect. Seven hypotheses were proposed, and a model for the concepts was built (see Figure 2). The solid lines in the figure indicate the five direct relationships hypothesized, and the dashed lines indicate the two hypothesized mediation relationships. Among the Hypotheses, H1 and H5 have already been confirmed, while the theoretical Hypotheses 2, 3, and 4 and mediation effect Hypotheses 6 and 7 are the focus of this study.

Two research goals are proposed: first, to test the hypothesis that environmental interpretation can enrich public ecological flow experiences and have a positive effect on promoting environmental attitudes and responsible behaviors; second, based on questionnaire data, to use a structural equation model (SEM) to test the interaction among public visit motivation, ecological flow experience, environmental attitude, and responsible behavior.

## 3. Research Methods

### 3.1. Case Study

**Figure 3:**
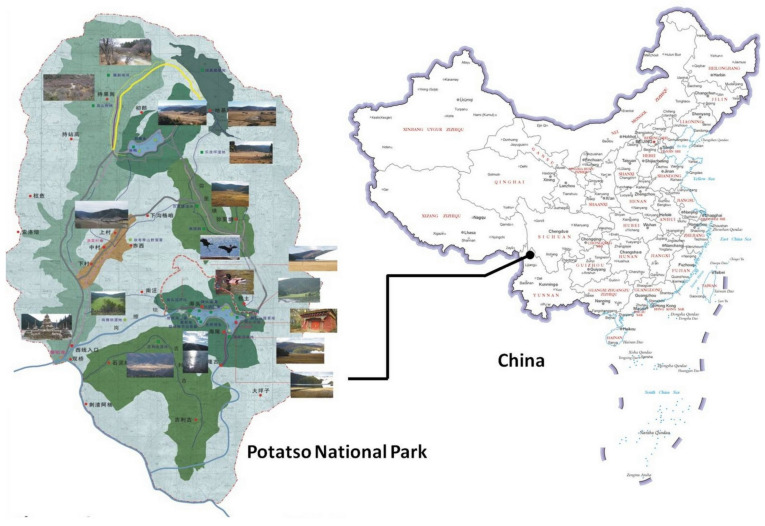
Geographical location of Potatso National Park.

Potatso National Park, Shangri-La, Yunnan Province, is located at the core hinterland of the “three parallel rivers” natural world heritage site, which is the location of one of the trails for China’s national park system (see Figure 3). The park covers a total area of 602.1 square kilometers, combining mountain meadows, lakes, snow-covered mountains, primeval forests, and geological relics. It features folk customs, a distinctive religious culture, biodiversity, landscape diversity, and cultural diversity as a whole, and is famous for its uniqueness, which gives the park a very high protection and display value.

There are two reasons Potatso National Park was chosen for the case study. First, the planning and building of environmental interpretation in the park started early around 2006, and the related facilities and services have been gradually implemented. Various media have been included in the park at present, and the public has praised the available service. Environmental interpretation in the park involves both personnel and non-personnel interpretation. Personnel interpretation mainly appears in the ecological science exhibition area and along the shuttle bus trail, including pictures, sand tables, freehand sketching, video, and real-item reproductions. Non-personnel interpretation includes visitor centers, interpretive panels, narrative brochures, videos, navigation maps, and smartphone applications. The visitor center provides easy-travel brochures, which concentrates the content of other narrative brochures, including public notices, a park overview, traffic advice, information on Lake Shudu lake, Militang, Bitahai lake, recommendations for visiting in the four different seasons, and an introduction to the plants and animals in the park. All kinds of signs in the park are relatively complete, with video and audio commentary always on display, and the studio is regularly open. Second, after a preliminary investigation, Potatso National Park opened Lake Shudu to the public starting on 3 September 2017, and to enhance public experience, five new routes were built around the lake. Under this condition of limited open resources, making full use of environmental interpretation to support natural education, promote the public ecological experience, and guide responsible behavior has become the key challenge and goal of Potatso national park.

### 3.2. Questionnaire Design and Distribution

The questionnaire design was divided into three stages. In stage one, the test questionnaire was designed and distributed, which yielded the initial questionnaire after an initial adjustment to and deletion of some of the original questions. After different routes were investigated and visitors were interviewed, the questionnaire was then adjusted a second time. Three managers from Potatso National Park and three scholars in the field were then invited to analyze the suitability of the questions. In stage two, 100 copies of the revised questionnaire were distributed and collected in the park in a pilot study. Based on the actual feedback from the public, the content of the questionnaire was further revised and the final version of the questionnaire was established. In stage three, the four-month questionnaire survey was launched. In addition, three scholars who specializing in environmental interpretation and park recreation were invited to revise and improve items of the questionnaires.

The questions were grouped into two parts. The first part included visit motivation, ecological flow experience, environmental attitude, and responsible behavior, with 47 questions in total. A third of the items were drawn from the existing literature (see Table 1). Other choices, especially those choices referring to environmental interpretation, were designed according to the actual conditions in the park. The second part gathered information on nine demographic characteristics: gender, age, residence, province, education, nationality, religion, career, and income. Questions on the visit characteristics were also included, including the mode of visiting, time of visit, whether the respondent has ever worked as a volunteer, how many visits have been made, and whether the respondent has visited other national parks. The questions were answered on a five-point Likert scale, ranging from 1, fully consistent, to 5, completely inconsistent.

### 3.3. Data Collection

The questionnaire was conducted with visitors of Potatso National Park via convenience sampling. Two points were set up in the park and at the shopping rest area outside the park for questionnaire collection. The respondents were sampled through a systematic sampling method that chose one out of every 20 visitors. The questionnaire was distributed to any member of the public who was willing to fill out the questionnaire; all respondents were given appropriate instructions. Completed questionnaires were collected on site. Visitors who were members of a group should not take up more than one-third of the total number of respondents. As Potatso is located in the Tibetan Plateau, where it is snowy during the other months of the year, visitors are not allowed to enter the national park from October to May. The survey lasted from June to September 2019, when visitors were allowed to enter the national park. The visiting hours for Potatso National Park are 9:00 a.m. to 4:00 p.m. The average time spent in the park varies between 1 and 3 h depending on the route chosen. The survey period was therefore set as: 10:30–12:30 a.m. and 14:00–18:00 p.m. July and August form the peak season, during summer vacation. In total, 750 questionnaires were distributed, and 630 were actually collected. A total of 568 questionnaires were valid, with an effective response rate of 90% (see Table 2).

## 4. Research Result and Analysis

### 4.1. Reliability and Validity Analysis

#### 4.1.1. Reliability Analysis

As shown in Table 3, the data from the scale of how environmental interpretation in the national park promotes public experience and responsible behavior met the ideal fitting standard, and the overall fit is good, which proves that the division into four dimensions is reasonable, the design of measurement items is reasonable and effective, and the internal consistency of the scale is high. The necessary conditions for validity analysis are thus met.

#### 4.1.2. Validity Analysis

For confirmatory factor analysis (CFA) to test the four dimensions, see Table 4. All dimensions have a composite reliability (CR) between 0.6 and 0.8; when CR is larger than 0.6, the validity is believed to be high. The standard level for factor loading is >0.5; the ideal figure is >0.7, and 75% of factor loadings in the parameter list are higher than 0.7, showing good aggregation among the four dimensions. All dimensions have an average variance extracted (AVE) higher than 0.5, indicating good convergent validity. The validity for the four dimensions meets the standards for research.

As shown in Table 5, the standard load coefficients for all items are between 0.5 and 0.91, showing good aggregation, so each item reflects its dimension well. It is generally considered reliable when the Cronbach’s alpha is higher than 0.6, and according to the table, these are from 0.69 to 0.9 for all items, while all items have a CR from 0.7 to 0.9, indicating high reliability for the items. For five items, the AVE is greater than 0.4, which is rather low, and three are between 0.55 and 0.92, but the AVE of the second order variables is higher than 0.5 and shows a fairly good fit to the model. The validity level of each item in the judgment scale meets the research requirements.

### 4.2. Theoretical Hypothesis Verification

#### 4.2.1. Model Fit Test

**Figure 4:**
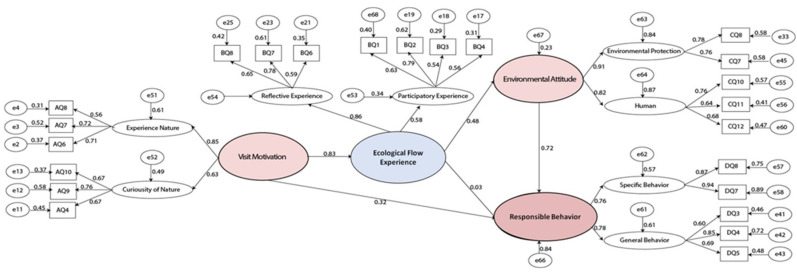
Final structural model.

The structural equation model of second-order factors was established according to the theoretical model for the influence process of national park environmental interpretation on the promotion of public experience and responsible behavior. The data from the 568 valid questionnaires were imported into the model, and standardized path coefficients were calculated, as shown in Figure 4.

Experience nature (standardized path coefficient 0.85) is the most significant factor for visit motivation, and curiosity about nature is the second. The stronger the motivation to experience nature, the more willing the public is to appreciate the mysterious primeval forest scenery and breathe unpolluted air, so they are thus more likely visit the park. The more curious they are about nature, the more willing they are to watch the lives of herdsmen who live in harmony with nature and observe rare animals and plants on the plateau.

Reflective experience (standardized path coefficient 0.86) is the most significant factor for ecological experience, and participatory experience is the second. The higher the efficiency of environmental interpretation, the more obvious the public’s experience of the beautiful scenery, precious resources, new knowledge, and the harmony between humanity and ecology in the national park. The higher the usage of environmental interpretation, the more the public can gain related to national park geology and geomorphology, biodiversity information, Tibetan culture and religion, and other physical geographical and human geographical knowledge.

Environmental protection (standardized path coefficient 0.91) is the most significant factor for responsible behavior, and specific behavior is the second. Not littering or picking plants in national parks are the most notable examples of responsible behavior. The public has become more sensitive to actions that damage the environment, and their willingness to promote the protection of wildlife and encourage others to act responsibly has increased significantly.

As shown in Table 6, the overall fit of the final model is acceptable, as shown by the following criteria: goodness of fit index (GFI) = 0.919, adjusted goodness of fit index (AGFI) = 0.898 ≈ 0.9, normed fit index (NFI) = 0.885 ≈ 0.9, Tucker–Lewis index TLI = 0.921, comparative fit index (CFI) = 0.932; three of these are higher than the average requirements, showing that the model obtained is ideal and has a good degree of fit. Further demonstration should not separate the path coefficients for visit motivation, ecological experience, environmental attitude, and responsible behavior. Rather, public visit motivation should join curiosity about nature, in combination with their willingness to experience nature; public ecological experience should be formed with participatory experience combining reflective experience. Public environmental attitude should be formed with environmental protection combining human development, and public responsible behavior should be formed with specific behavior combining general behavior.

#### 4.2.2. Direct Effect Test

The standardized path coefficients were calculated by importing the valid questionnaire data. The interactions among visit motivation, ecological flow experience, environmental attitude, and responsible behavior were tested, as shown in Table 7. Hypothesis 1, 4 and 5 were supported, while Hypothesis 2 and 3 were not.

Hypothesis 1 is supported: visit motivation has a significant and positive effect on ecological flow experience. The estimated path coefficient is 0.473, indicating that an increase of visiting motivation by 1 unit can directly promote the improvement of environmental interpretation of the ecological flow experience by 0.473 units.

Hypothesis 2 is not supported: the *p*-value is 0.147, and the estimated path coefficient is 0.256, showing that visit motivation does not have a significant impact on public responsible behavior, which means that visit motivation does not directly affect public responsible behavior.

Hypothesis 3 is not supported: the *p*-value is 0.88, which is greater than 0.05, and the estimated path coefficient is 0.037, showing that ecological flow experience does not have a significant impact on public responsible behavior, which means that ecological flow experience does not directly affect public responsible behavior.

Hypothesis 4 is supported: the *p*-value is smaller than 0.05, and the estimated path coefficient is 0.773, indicating that ecological flow experience has a significant and positive effect on public environmental attitude, and an increase of 1 unit of ecological flow experience can directly improve environmental attitude by 0.773 units.

Hypothesis 5 is supported: public environmental attitude has a significant and positive effect on responsible behavior. The estimated path coefficient is 0.635, indicating that a 1-unit increase in environmental attitude promotes an increase of 0.635 units of responsible behavior.

#### 4.2.3. Mediating Effect Test

This study hypothesized two mediating effects: Hypothesis 6, ecological flow experience mediates the influence of visit motivation on responsible behavior; and Hypothesis 7, ecological flow experience mediates the influence of visit motivation on environmental attitudes and guiding responsible behaviors. The analysis verified that Hypothesis 6 is not supported, while Hypothesis 7 is supported, as shown in Table 8.

Hypothesis 6 is not supported: the *p*-value is 0.903, indicating ecological flow experience cannot be a mediating variable for the influence of visit motivation on responsible behavior. In other words, visit motivation does not influence public responsible behavior through ecological experience.

Hypothesis 7 is supported: the *p* value is 0.000, which is smaller than 0.05. The mediating effect path is valid, and the ecological flow experience is the mediating variable for the influence of visit motivation on environmental attitude and responsible behavior. This conclusion proves that, under the influence of visit motivation, environmental interpretation in the national park is necessary and indispensable to enrich the public ecological experience and stimulate the public’s environmental attitude and responsible behavior. Overall, environmental interpretation enriches the public ecological flow experience, leading to changes in environmental attitude, which in turn affects the public’s responsible behavior.

## 5. Discussion and Conclusions

### 5.1. Discussion

According to the survey, the public thinks that environmental interpretation is important, and they are willing to engage with it as a service. Managers should consider personnel and non-personnel environmental interpretation mediums. To further enrich the public ecological experience, the park should promote the effective use of APP, integrating manuals, environmental interpretation content from popular science areas, and interpretative activities, as well as condensing the theme on the structure of nature, humanity, and behavior. This would allow the deepening of the interpretative focus on how human activities affect the earth’s ecological environment and sustainable development, which would promote the formation of public environmental attitudes in a multi-dimensional way within a limited time and space. Park managers should strengthen the cultivation of public ecological awareness, as well as the distinction between environmentally damaging and responsible behaviors. The research suggest that managers could promote a public platform that is regularly updated with short videos and pictures to publicize activities for science popularization and environmental education. This could include, for example, appealing wildlife scenes (e.g., staff in Potatso National Park helped a wild bear living in a primitive forest in August 2019) or interesting videos in the park. This would create positive memories and provide the public a chance to reflect and establish place attachment. Park managers should also define the spatial dimension structures of point, line, plane, and net, and refine the time dimension structures of peak and low peak periods to better oversee public visits.

### 5.2. Limitation

Field work and a survey were carried out in Potatso National Park in this study, and the data were analyzed using structural equation modeling in AMOS software. Important results were obtained, but there are still some areas that could be further improved. First, the survey was carried out during one year, and a comprehensive survey of the public in several years may be sound to understand visitors’ motivation and experience. Second, reduced space was available for visiting because Potatso National Park was in a pilot reform period and had been affected by COVID-19, and some interpretive points were closed at the time of the survey. The service scope of environmental interpretation was passively reduced, and the opportunity for ecological experience was slightly decreased. Future studies could undertake public surveys in different seasons to track the upgrading of the environmental interpretation facilities and services over a wider area, providing a valuable reference for the construction of Potatso National Park. Third, similar studies should be conducted on environmental interpretation in other national parks, and the moderating effects of different classification variables on the promotion of public ecological experiences and responsible behavior should be analyzed.

### 5.3. Conclusions

This study proposes seven theoretical hypotheses and establishes theoretical models of the variables of visiting motivation, ecological flow experience, environmental attitude, and responsible behavior, under the premise that environmental interpretation in a national park is in effect. The data were collected from 568 visitors at Potatso National Park during four months in 2019. The structural equation modeling findings reveal the key factors and effect paths of promoting visitors’ ecological flow experience and impact on environmental attitude and responsible behavior at the national park. The results presented the total impact on the promotion of the public’s responsible behavior including the direct impact and indirect impact. Based on the above analysis, we could reach three conclusions.

First, visit motivation directly improves the ecological flow experience, but does not directly stimulate responsible behavior. The public have obvious motivation to visit based on a desire to experience the natural views and culture in the national parks, and they have a clear intention to experience the primitive forest scenery and local Tibetan life in Potatso National Park. Some visitors come with tour groups, some come because of the recommendation of their relatives, and some know about the park through media. The motivation to visit is a direct driver for them to find more channels for experience, thus yielding opportunities to enrich their own ecological experience. Visit motivation alone cannot, however, fully promote responsible behavior, but can only be treated as a prerequisite. This study suggests that the managers of Potatso National Park pay more attention to the public’s experience needs and desires, and provide more diversified and secure channels for the public to enjoy the plateau’s virgin forest scenery. Tibetan villagers should be fully motivated to show their true lifeways to the public. The national park should try to focus on the plateau’s primitive forest scenery and the living conditions of the Tibetans, while inviting the public to experience the deeply combined natural and cultural scenery.

Second, environmental interpretation plays a significant role in enriching the public ecological experience and guiding attitudes about environmental protection. Environmental interpretation conveys scientific knowledge, on topics such as geography, ecology, and history, as well as humanistic knowledge, in areas such as nationality, culture, and religion, to satisfy public curiosity and reflect the human impact on the ecological flow environment. It can also make the public realize the significance of environmental protection and encourage the public’s thoughts about human development. The service also guides the public to realize the difficulty of maintaining the beautiful landscape and being actively integrated into the ecological environment. Gaining knowledge and enjoying the physical and emotional enjoyment of beautiful scenery stimulates the public’s emotions and makes them feel the charm of the oriental philosophy of harmony between man and ecology, thus enriching their visit to the national park.

Third, visit motivation promotes ecological flow experience, and ecological flow experience affects environmental attitude, ultimately leading to responsible behavior. Environmental interpretation stimulates environmental attitudes and guides responsible behavior by enriching the public’s ecological experience. The shift in environmental attitude can be seen directly in the public consciously protecting the environment in the park (e.g., by not throwing trash, not picking plants, and not harming wild animals). It can appear indirectly as the public’s acute awareness of environmentally damaging and responsible behavior, and a desire to protect wildlife. In other words, “environmental attitude” is the determining factor for whether the public engages in responsible behavior. If this attitude is not changed, responsible behavior will not appear. Environmental interpretation is an effective method to enrich the public’s ecological flow experience and promote the necessary shift in environmental attitudes, which is an important discovery of the present research.

In conclusion, environmental interpretation in national parks plays an important role in enriching the public’s ecological flow experience. The service is thus important and necessary for arousing the public’s environmental attitude and spurring responsible behavior. Such environmental interpretation should thus be established as the main method for promoting the public’s positive environmental attitude, strengthening cooperation among organizations, adding natural education programs, and providing environmental knowledge to the public. In view of the lifting of restrictions at the park, strengthening the environmental interpretation facilities and methods on the newly added routes and adjusting the configuration of the existing interpretation media in the three-dimensional structure of theme, space, and time are recommended. This research provides evidence that environmental interpretation in national parks plays an indispensable role in promoting the ecological flow experiences and responsible behavior of the public.

## Figures and Tables

**Table 1:** Reference list for questionnaire items.

Reference Author	Reference Choice
ZHAO Minyan (2019) [2]	AQ12 Learn about the natural environment
	AQ15 Socialize
LIU Chuanan (2016) [53]	AQ13 Enhance emotional communication with relatives and friends
SONG Qiu (2008) [5]	AQ14 Work requirement
LIU Weifeng (2011) [54]	CQ1 More environmental interpretations are important
	CQ6 Polluting the environment is immoral
	CQ8 Biodiversity and wildlife depend on human awareness of environmental protection
	DQ1 I am willing to participate in environmental interpretation
	DQ7 I will not throw trash in the park
YU Yong (2010) [55]	CQ2 The ecological environment is vulnerable
	CQ3 Humanity is part of the natural ecological environment
LUO Fen (2011) [56]	CQ4 Human activities do not have much effect on the natural environment
HONG Xueting (2018) [34]	CQ5 Animals, plants, and humans can coexist harmoniously in nature
LI Hongjun (2018) [57]	DQ4 I will encourage others to take actions that are good for the park environment
DONG Xin (2018) [58]	DQ10 I will not smoke in non-smoking areas of national parks

**Table 2:** Sample demographic characteristics.

Item	Category	Number	Percentage (%)	Item	Category	Number	Percentage (%)
**Gender**	Male	228	40.1	**Career**	Student	224	39.4
	Female	340	59.9		Teacher/Technician	94	16.6
**Age**	18 and below	146	25.7		Official	28	4.9
	19–30	193	34		Public service	47	8.3
	31–45	172	30.3		Company employee	93	16.4
	46–60	50	8.8		Farmer/worker	15	2.6
	More than 60	7	1.2		Retired	7	1.2
**Residence**	Big cities	238	41.9		Other	60	10.6
	Small and medium-sized cities	291	51.2	**Income**	<3000	74	13
	Rural area	39	6.9		3000–5000	211	37.1
**Province**	Yunnan	64	11.3		5000–10,000	146	25.7
	Outer Yunnan	470	82.8		10,000–15,000	97	17.1
	Overseas	34	5.9		15,000–20,000	14	2.5
**Education**	Junior high and below	123	21.6		>20,000	26	4.6
	Senior high/Secondary school	94	16.6	**Time of travel**	Winter/summer vacation	408	71.8
	Junior college/Undergraduate	291	51.2		Labor Day holiday	38	6.7
	Postgraduate and above	60	10.6		National Day holiday	30	5.3
**Nationality**	Han	438	77.1		Spring Festival	20	3.5
	Tibetan	10	1.8		Other	72	12.7
	Other	120	21.1	**Mode of travel**	Alone	42	7.4
**Religion**	Buddhism	102	18		With family	357	62.8
	Christian	10	1.7		With colleagues	118	20.8
	Other	17	3		Package tour	51	9
	None	439	77.3	**Ever visited other national parks**	Yes	448	78.9
**Number of visits**	The first time	506	89.1		No	120	21.1
	The second time	57	10	**Ever worked as a volunteer**	Yes	201	35.4
	More than twice	5	0.9		No	367	64.6

**Table 3:** Overall adaptability of the scale.

Fitness Index	c2/df	GFI	AGFI	NFI	TLI	CFI	RMSEA	SRMR
Fitting standard	≤3	≥0.90	≥0.90	≥0.90	≥0.90	≥0.90	≤0.08	≤0.05
Test model	2.245	0.908	0.886	0.85	0.897	0.91	0.05	0.051
Fitting situation	Ideal	Ideal	Fairly ideal	Fairly ideal	Fairly ideal	Ideal	Ideal	Fairly ideal

Note: goodness of fit index (GFI), adjusted goodness of fit index (AGFI), normed fit index (NFI), Tucker–Lewis index (TLI), root mean square error approximation (RMSEA), standardized root mean square residual (SRMR).

**Table 4:** Confirmatory factor analysis of four dimensions.

Dimension	Parameter	Standard Load Coefficient	Reliability	Cronbach’s Alpha	CR	AVE
Visit motivation	Curiosity of nature	0.626	0.392	0.742	0.709	0.555
	Experience nature	0.847	0.717			
Ecological experience flow	Participatory experience	0.580	0.336	0.699	0.695	0.542
	Reflective experience	0.865	0.748			
Environmental attitude	Human	0.823	0.677			
	Environmental protection	0.912	0.832	0.791	0.860	0.755
Responsible behavior	General behavior	0.776	0.602			
	Specific behavior	0.768	0.590	0.819	0.747	0.596

Note: Composite reliability (CR); average variance extracted (AVE).

**Table 5:** Parameters of item confirmatory factor analysis.

Parameter	Standard Load Coefficient	Reliability	Cronbach’s Alpha	CR	AVE
**Curiosity of nature**			**0.716**	**0.722**	**0.467**
How Tibetan herdsmen make a living in the park	0.669	0.448			
Watch life scenes of Tibetan herdsmen	0.762	0.581			
Observe unique plateau animals and plants	0.609	0.371			
**Experience nature**			**0.692**	**0.706**	**0.448**
Enjoy mysterious highland scenery	0.713	0.508			
Experience primeval forest on plateau	0.725	0.526			
Feel the virgin forest without pollution	0.556	0.309			
**Participatory experience**			**0.718**	**0.727**	**0.405**
The explanatory brochure gave me an overview of Potatso (geology, flora and fauna, ecosystem, transportation, scenic spot information)	0.629	0.396			
Popular science helped me understand how the park formed and evolved, biodiversity information, traditional Tibetan cultural life, and religious ideas	0.788	0.621			
Interpreters introduced the functions and development of the national park, the concept of protection, the meaning of the name of the scenic spot and resource types, animal and plant information, fairy tales, and tour routes	0.540	0.292			
The explanatory board informed me about the scenic area animal and plant information, route instructions, scenic spot overview, guide protection behavior, and environmental knowledge	0.558	0.311			
**Reflective experience**			**0.706**	**0.719**	**0.464**
The park has beautiful scenery and precious resources	0.594	0.353			
The new knowledge satisfied my curiosity. I was impressed by the ecological environment and biological information in the park	0.783	0.613			
I have merged into nature, and feel the harmony between humanity and ecology	0.652	0.425			
**Human**			**0.731**	**0.737**	**0.484**
Environmental awareness promotes sustainable development	0.755	0.570			
Environmental pollution affects human development	0.644	0.415			
Destroying the environment is not good for human development	0.683	0.466			
**Environmental protection**			**0.734**	**0.736**	**0.582**
Primeval forests need to be protected	0.765	0.585			
Human awareness of environmental protection is conducive to promoting biodiversity and protecting wildlife	0.761	0.579			
**General behavior**			**0.749**	**0.786**	**0.553**
I am aware of people around me destroying the environment	0.676	0.457			
I encourage others to take actions that are good for the environment	0.849	0.721			
I am willing to promote wildlife protection to others	0.693	0.480			
**Specific behavior**			**0.9**	**0.904**	**0.826**
I won’t throw litter in the park	0.911	0.830			
I won’t hurt the plants in the park	0.906	0.821			

**Table 6:** Overall fitting index of the second-order factor model.

Fitting Index	c2/df	GFI	AGFI	NFI	TLI	CFI	RMSEA	SRMR
Fitting standard	≤3	≥0.90	≥0.90	≥0.90	≥0.90	≥0.90	≤0.08	≤0.05
Test model	2.264	0.919	0.898	0.885	0.921	0.932	0.05	0.057
Fitting condition	Ideal	Ideal	Fairly ideal	Fairly ideal	Ideal	Ideal	Ideal	Fairly ideal

Note: goodness of fit index (GFI), adjusted goodness of fit index (AGFI), normed fit index (NFI), Tucker–Lewis index (TLI), root mean square error approximation (RMSEA), standardized root mean square residual (SRMR).

**Table 7:** Second-order factor model normalization path.

Hypothesis	Estimate	SE	CR	*p*	Result
**Hypothesis 1 (H1).** *Visitmotivation will havea positive effect onecologicalflowexperience*.	0.473	0.084	5.631	***	True
**Hypothesis 2 (H2).***Visitmotivation willhavea positive effect onresponsible behavior*.	0.256	0.362	0.153	0.147	False
**Hypothesis 3 (H3).** *Ecologicalflowexperience willhavea positive influence onresponsible behavior*.	0.037	0.248	0.141	0.88	False
**Hypothesis 4 (H4).** *Ecologicalflowexperience willhavea positive effect onenvironmental attitude*.	0.773	0.137	5.651	***	True
**Hypothesis 5 (H5).** *Environmental attitude willhavea positive effect onresponsible behavior*.	0.635	0.081	7.818	***	True

Note: Standard error (SE),Critical ration (CR), Significance (P), *p* < 0.001 (***).

**Table 8:** Intermediate effect test.

	Estimate	SE	Lower	Upper	*p*	Result
**Hypothesis 6 (** **H6).** *Visit motivation—ecological flow experience—responsible behavior.*	0.018	0.331	−0.631	0.417	0.903	False
**Hypothesis 7 (** **H7).** *Visit motivation—ecological flow experience—environmental attitude—responsible behavior.*	0.232	0.074	0.137	0.442	0.000	True

Note: Standard error (SE), significance (P).

## Data Availability

The data presented in this study are available on request from the corresponding authors.
